# Pilot early phase II study of decitabine and carboplatin in patients with advanced melanoma

**DOI:** 10.1097/MD.0000000000020705

**Published:** 2020-06-19

**Authors:** Andre van der Westhuizen, Naomi Knoblauch, Moira C. Graves, Richard Levy, Ricardo E. Vilain, Nikola A. Bowden

**Affiliations:** a Department of Medical Oncology, Calvary Mater Hospital, Newcastle; b Centre for Drug Repurposing and Medicines Research, School of Medicine and Public Health, University of Newcastle and Hunter Medical Research Institute; c Department of Surgical Oncology, Calvary Mater Hospital; d Department of Anatomical Pathology, Pathology North, NSW Health Pathology, Newcastle, NSW, Australia.

**Keywords:** carboplatin, decitabine, epigenetics, immunotherapy resistance, melanoma

## Abstract

**Introduction:**

Resistance to targeted and immune checkpoint blockade treatment remains a major problem in patients with advanced metastatic melanoma. To overcome this problem, there needs to be a decrease in burden of disease as well as re-establishing of immune sensitivity. The aim of this early phase 2 clinical trial is to investigate a novel way of sequencing and combining decitabine and carboplatin to decrease methylation and increase DNA repair resulting in a decrease in the disease burden and to re-establish sensitivity to the immune response.

**Methods and analysis:**

This single-site early phase 2 clinical trial will be conducted in 30 patients with metastatic melanoma that are resistant to all approved therapies. Patients will receive 2 × 4-week cycles of decitabine 7 mg/m^2^ IVI/day for 5 days (D1-D5) followed by Carboplatin AUC 5 IVI on D8; Week 3 and Week 4 no treatment. The primary objective is to determine DNA methylation and DNA repair levels before, and immediately after treatment; quantify immune-response markers (PDL-1, PD-1, CD4/CD8, and CD68) in blood, tumor and microenvironment before treatment and after 2 cycles. The secondary outcome objective is to quantify response rate (RR) to administration of 2 cycles of decitabine and carboplatin cycle using response evaluation criteria in solid tumors (RECIST 1.1) criteria. This data will be used to calculate sample size and determine statistical analysis plan for larger Phase 2 study.

**Ethics and dissemination:**

Protocol version 1.1 was reviewed and approved by the Hunter New England Health Human Research Ethics Committee (Reference No: 15/12/16/3.08, NSW HREC Reference No: HREC/15/HNE/505) and site-specific approval from the Calvary Mater Hospital Newcastle, NSW, Australia (NSW SSA Reference No: SSA/16/HNE/224). Primary and secondary outcomes and safety data will be disseminated through publications.

**Trial registration details:**

Australian New Zealand Clinical Trial Registry ACTRN12616000440426

**Article summary**

- This is a new combination of drugs for the treatment of metastatic melanoma to determine if the tumor becomes immunogenic in order for it to be used as a priming regime in combination with immunotherapy in future.
- Early phase II, open label single cohort study of 30 patients at single site (Calvary Mater Hospital Newcastle, Australia)
- This early phase II study will assess the feasibility of a larger Phase II study and determine outcome measures required to calculate sample sizes and develop a statistical plan.

## 1 Introduction

Resistance to targeted and immune checkpoint blockade treatment remains a major problem in patients with advanced metastatic melanoma. Melanoma patients with advanced disease, especially after targeted therapy, do not respond as well to immune therapy because of high disease burden and the development of immune therapy resistance.^[1]^ To overcome this problem, there needs to be a decrease in burden of disease as well as re-establishing of immune sensitivity. The aim of this early phase 2 clinical trial is to investigate a novel way of sequencing and combining agents to decrease the disease burden and to re-establish sensitivity to the immune response.

Platinum chemotherapy is used to treat most solid tumors, with germ-cell tumors obtaining cure rates of over 90% when treated with cisplatin. Despite this, it has limited efficacy in melanoma (∼10%) and as a consequence is rarely used in treatment. The mechanism of action for cisplatin and its analogue carboplatin, is insertion of platinum into DNA to form cross-links which is structurally very similar to UV-induced DNA damage. Recognition of DNA cross-links by the global genome repair proteins XPC, DNA-damage binding protein 1 and DNA-damage binding protein 2 triggers apoptosis rather than attempting to repair the damage. Therefore, reduced levels of global genome repair result in a lack of apoptotic signaling, leading to limited or no response to platinum treatment and an increase in “UV-like” mutation load across the platinum-treated genome.

5-aza-2’-deoxycytidine (decitabine) reactivates the expression of genes silenced by hypermethylation. Decitabine is a cytidine analogue that incorporates into replicating DNA and blocks the action of DNA methyltransferases. Previous studies have shown that decitabine enhances the anti-tumor immune response and at low dosage correlates with clinical response in hematological malignancies.

We previously reported very low levels of XPC, DNA-damage binding protein 1, and DNA-damage binding protein 2 in melanoma cell lines that are not inducible by carboplatin or its analogue, cisplatin.^[2,3]^ Subsequent analysis of advanced primary and metastatic melanomas from the Hunter region, NSW, Australia and the Cancer Genome Atlas (TCGA) data from 378 melanomas identified very low levels of XPC expression.^[4]^ Analysis of XPC methylation using Illumina 450K methylation array data from the 378 TCGA melanomas. A region -3000 bp upstream of XPC was fully methylated (β > 0.6) in 125 melanomas (33.1%) and partially methylated (β 0.2–0.6) in 253 melanomas (66.9%). Methylation of the CpG dinucleotides within the proximal XPC promoter region (−1 to −175 bp upstream) was not detected in the TCGA cohort. Further to this, we have found melanoma cell lines contain methylation in the CpG island shores that can be removed via blocking of methyltransferases with decitabine.^[5]^

In pre-clinical studies melanoma cell lines were treated with 0.26 uM decitabine for 72 hours to reduce methylation across the genome, followed by 8 ug/mL carboplatin for 48 hours to induce DNA damage, apoptosis and cellular stress. The doses used were the lowest clinically relevant doses to induce a response. The majority of melanoma cell lines showed an induction of XPC (range = 1.1–3.2 fold increase) and increased apoptotic cell death after the decitabine/carboplatin sequential treatment compared to carboplatin alone (range = 1.6–2.2 fold increase). Only one melanoma cell line showed no increase in XPC or apoptosis after the sequential combination treatment, but did express markers of senescence. All cells lines displayed a significant decrease in cell proliferation after the sequential combination.^[5]^

There is established evidence for safety of decitabine/carboplatin sequential treatment. A phase I dose escalation study in platinum-resistant ovarian cancer reported no dose-limiting toxicity at 10 mg/m^2^ decitibine for 5 days followed by AUC5 carboplatin on Day 8 of a 28 day cycle.^[6]^ This dose regime was repeated in a larger cohort (n = 17) and the most common toxicities were nausea (n = 10), constipation (n = 7), allergic reactions (n = 6), neutropenia, fatigue, anemia (5 patients each), the majority being grades 1–2. Grade 3–4 toxicities affecting more than one patient included neutropenia (n = 4) and thrombocytopenia (n = 2).^[7]^ Therefore, to minimize toxicity further, this protocol uses a lower dose regime of decitabine 7 mg/m^2^ for 5 days followed by Carboplatin AUC5 on day 8. AUC5 of carboplatin is the only dosage previously tested in sequential combination with decitabine^[6,7]^ and to ensure minimal toxicity it is AUC1 lower than the most recent clinical trial outcomes that included carboplatin treatment of melanoma.^[8]^

The primary hypothesis for this protocol is that sequential treatment with decitabine and carboplatin will result in:

i) decrease in methylation and increase in XPC in tumors
ii) increase in immune response markers in tumor and blood.

The secondary hypothesis is that sequential treatment with decitabine and carboplatin will induce an overall response of reduction of tumor size or number (assessed by RECIST) or stable disease indicated by no increase in tumor size or number (assessed by RECIST).

## 2 Methods and analysis

### 2.1 Study objectives

#### 2.1.1 Primary outcome objectives

1. Determine DNA methylation and XPC levels before, and immediately after treatment, that is, at study entry and after 2 months.
2. Quantify immune-response markers (PDL-1, PD-1, CD4/CD8, and CD68) in blood, tumor and microenvironment and immune response in blood before treatment and after 2 cycles.

#### 2.1.2 Secondary outcome objectives

1. Quantify response rate (RR) to administration of 2 cycles of decitabine and carboplatin/28 day cycle using RECIST criteria. This data will be used to calculate sample size and determine statistical analysis plan for larger Phase II study.

This is a single-center study conducted at Calvary Mater Hospital, Newcastle NSW Australia in a single group of 30 patients. Surgical biopsies will be conducted as day surgery and all participants will receive treatment as day oncology patients. There will be no requirement for participants to be admitted to hospital.

### 2.2 Treatment protocol

Patients will receive a four week treatment protocol consisting of Decitabine 7 mg/m^2^ IVI/day for 5 days (D1-D5) followed by Carboplatin AUC 5 IVI on Day 8 (D8). Week 3 and Week 4 there is no treatment (Table 1). This four week protocol is repeated once starting at Week 5, Day 29 (Table 1).

**Table 1:**
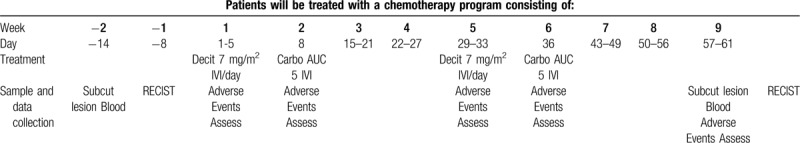
Study outline.

Adverse events will be assessed on Day 1, Day 8, Day 29, Day 36, and Day 57 (Table 1) and graded according to common terminology Criteria for AEs, National Cancer Institute (NCI-CTCAE) v4.

### 2.3 Data collection

Before treatment (Day -14) and on Day 57 (week 9) an excision biopsy of subcutaneous metastatic lesion or lymph node and 9 mL blood collection will be collected where. Tumor biopsies will be used for analysis of DNA methylation (whole genome bisulfite sequencing and ELISA assay), XPC mRNA and protein levels. Statistically significant differences in methylation and XPC levels will be tested before and after treatment using paired *t* test with Bonferroni correction.

Tumor biopsies will also be used for immunohistochemistry to assess the immune-response markers: CD4 and CD8 inside tumor, number of tumor cells with PDL1 expression, CD8 with PDL1 expression, CD8 with CD45RO expression, CD8 with granzyme B, TIM3, perforin as described in Tumeh et al.^[9]^ Blood collected will be tested for immune activation profile and INF gamma signature. All data will be tested before and after treatment using paired t-test with Bonferroni correction.

#### 2.3.1 Secondary outcome protocol

Overall response rate (ORR) will be determined using RECIST 1.1 criteria at completion of the 2 treatment cycles in week 9. This data will be used to calculate sample size for larger Phase II study.

### 2.4 Inclusion criteria, screening procedures and pre-treatment investigations

If laboratory investigations have not been performed within the 7 day period prior to starting treatment then they must be repeated prior to day 1 of cycle 1 to ensure the patient is fit to receive platinum based chemotherapy and Decitabine, this does not apply for endocrinology investigations (thyroid function test, adrenocorticotropic hormone, cortisol, s-testosterone) where these investigations can be performed within 21 days prior to starting treatment

Patients need to meet inclusion criteria outline in Table 2. Patients should have CT scans and positron emission tomography scans before treatment starts and the same investigations should be performed using the same provider after 2 cycles of treatment. Target and non-target lesions will be identified clinically and on CT scan (Table 2).

**Table 2:**
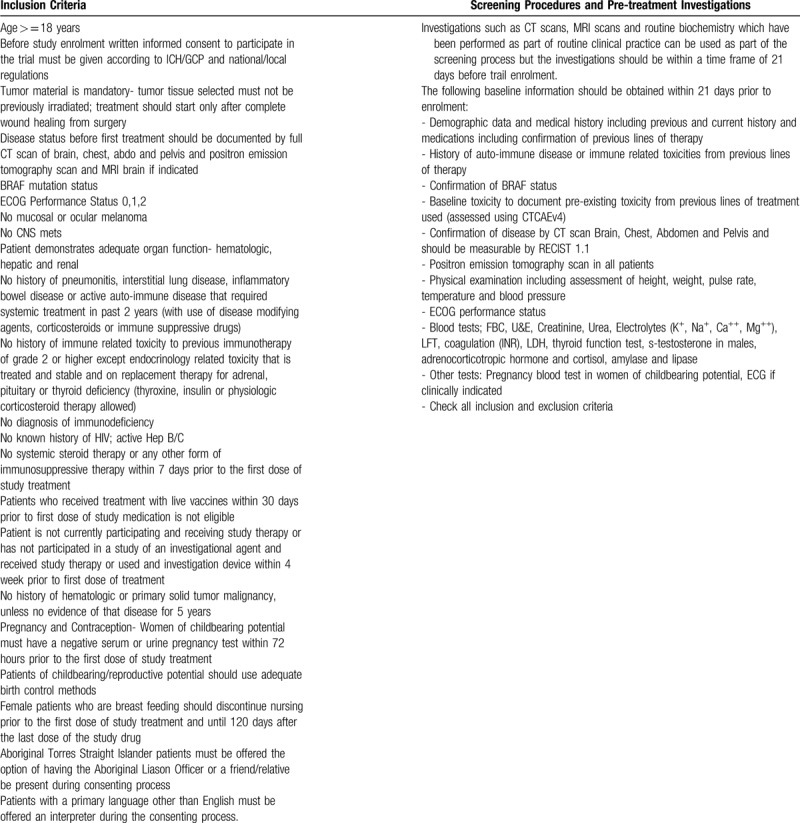
Inclusion criteria, screening procedures, and pre-treatment investigations.

Target AUC X (GFR + 25) for Carboplatin dose, for the purpose of this protocol the GFR is considered equivalent to the creatinine clearance. The GFR should be calculated as per local practice and the exact dose of Carboplatin depends on the GFR and the dose should be checked with the Medical Oncology Clinical Trial Pharmacist. If the estimated creatinine clearance is < 50 ml/minute, then a formal measurement of the GFR is required using an isotopic clearance and if the isotopic clearance is used then the value uncorrected for body surface area (BSA) should be used in dose calculations. If the GFR is measured by isotopic clearance, the target AUC is one unit lower than that based on the estimated GFR (4 and not 5).

The GFR should be recalculated for renal toxicity (serum creatinine > 1.5 upper limit of normal), serum creatinine changes of > 10% compared to baseline or last creatinine value used to calculate carboplatin dose (whichever is more recent), for each dose modification of carboplatin and cycle 2 if according to clinical judgment there has been significant doubt about the true GFR at cycle 1.

Dose capping of carboplatin should be done according to standard local practice and should be checked with the Medical Oncology Clinical Trial Pharmacist. The clinical judgment of an experienced clinician should be applied to calculate the Carboplatin dose as patients who have prolonged post-operative recovery and who have been maintained on prolonged IVI fluids with poor nutrition will have falsely low serum creatinine and formulae such as the Cockcroft-Gault formula for GFR are inaccurate at the extremes of age and weight.

### 2.5 Toxicity

*Hematological toxicity:* chemotherapy should be delayed if either of the following occurs in the pre-treatment blood count: absolute neutrophil count less than 1.5 × 10^9^/l; platelet count less than 100 × 10^9^/l. Full blood count should be repeated weekly until hematological recovery has occurred. If hematological recovery takes longer than 7 days the dose of Carboplatin should be reduced according to standard criteria by 1 AUC before cycle 2 is given.

Febrile neutropenia is defined as fever with or without clinically or microbiologically documented infection with ANC less than 1 × 10^9^/l and fever >  = 38.5 °C and this should lead to a dose modification of 1 AUC before cycle 2.

*Renal Toxicity*: As described above, the dose of Carboplatin should be recalculated for renal toxicity. There are no specific dose modifications for renal toxicity before cycle 2 and this combination of treatment is not directly expected to cause renal toxicity.

*Hypersensitivity:* A hypersensitivity reaction to Carboplatin should be managed according to standard local practice. Patients may be retreated according to local practice, including escalations of hypersensitivity prophylaxis, in-patient monitoring and increased in the duration of the infusion.

*Hepatotoxicity:* Hepatotoxicity is not expected with this combination.

*Other toxicities*: Nausea, constipation, fatigue, anorexia, diarrhea, vomiting and dizziness were reported as treatment related adverse events in a previous trial where this combination was used. These will be managed by standard local practice guidelines and will be graded according to severity. The majority of these toxicities were grade 1–2. The only grade 3–4 toxicities reported on this combination of treatment before that affected more than 1 patient included neutropenia and thrombocytopenia (management already discussed).

### 2.6 Sample size calculation

This is a signal-seeking Early Phase 2 study to inform sample size calculation for a larger Phase 2 study after completion. Power calculation based on incidence of response: Assuming ORR is <1% in untreated immunotherapy-resistant melanoma, using Simon 2-stage design, initial recruitment (n = 17) is 80% powered to detect ORR of 20% (α = 0.05). The second stage will be initiated if there is at least one response in the first 17 patients. In the second stage, 23 additional patients (n = 40) will be enrolled to achieve 80% power to detect 10% ORR (α = 0.05).

### 2.7 Data analysis plan

All data for primary outcomes will be tested before and after treatment using paired t-test with Bonferroni correction when required.

Objective response rate (ORR) is defined as the proportion of patients with tumor size reduction of 30% after onset of treatment until documented tumor progression. ORR will be calculated using RECIST scores at weeks −1 and 9. Before-after study (paired *t* test): RECIST scores will undergo paired t-test analysis to determine statistical differences in response based on tumor size. The results of the before-after study will be used to calculate the sample size for a large multi-center Phase 2 trial.

Waterfall plot of tumor shrinkage is a data visualization technique that depicts tumor shrinkage that is the best observed % change of sum of target lesions from baseline.

Overall survival will be calculated at each RECIST data collection point (weeks −1 and 9) and every 6 months until study completion. Overall survival will be calculated as the time from initiation of the trial treatment to death from any cause. Kaplan-Meier plots for sub-groups based on primary outcome data will be generated every 6 months for the duration of the study.

### 2.8 Ethics, safety, and dissemination

This protocol was reviewed and approved by the Hunter New England Health Human Research Ethics Committee (Reference No: 15/12/16/3.08, NSW HREC Reference No: HREC/15/HNE/505) and site-specific approval from the Calvary Mater Hospital Newcastle, NSW, Australia (NSW SSA Reference No: SSA/16/HNE/224).

Safety assessments will be done as outlined in the trial schema in Table 1. Medical judgment will be exercised in deciding whether an adverse event is serious. A serious adverse event is any adverse reaction or unexpected adverse reaction that: Results in death, is life threatening, requires hospitalization or prolongation of existing hospitalization, results in persistent or significant disability or incapacity, consists of congenital anomaly or birth defect, results in other important medical condition. The sponsor will determine if the trial is to terminate due to serious adverse events.

Important adverse events/reactions that are not immediately life threatening or do not result in death or hospitalization but may jeopardize the subject or may require intervention to prevent one of the outcomes listed in the definition above, should also be considered serious. The Investigator will assess the causality of all serious adverse events in relation to the trial therapy using standardized definitions of causality.

Primary and secondary outcomes and safety data will be disseminated through publications.

## Author contributions

AvdW devised the concept and drafted and approved the full protocol, NK is clinical trial coordinator for the protocol, MCG contributed to the protocol for assessing primary outcomes, RL contributed to the biopsy protocol, REV contributed to inclusion/exclusion criteria and primary outcomes protocol and NAB devised the concept, drafted and approved the full protocol.

**Conceptualization:** Nikola Bowden.

**Data curation:** Naomi Knoblauch.

**Formal analysis:** Moira Graves, Ricardo Vilain, Nikola Bowden.

**Funding acquisition:** Nikola Bowden.

**Investigation:** Moira Graves, Richard Levy, Nikola Bowden.

**Methodology:** Richard Levy, Ricardo Vilain, Nikola Bowden.

**Project administration:** Naomi Knoblauch, Nikola Bowden.

**Supervision:** Ricardo Vilain, Nikola Bowden.

**Writing – original draft:** Nikola Bowden.

**Writing – review & editing:** Ricardo Vilain, Nikola Bowden.
